# Effect of Temperature on Skipper Fly *Piophila casei* (Insect: Diptera) Reared on Ham †

**DOI:** 10.3390/insects16100993

**Published:** 2025-09-24

**Authors:** Annalisa Grisendi, Chiara Lucchetti, Mara Scremin, Mattia Calzolari, Deborah Torri, Federica Savini, Paolo Bonilauri, Michele Dottori

**Affiliations:** 1 Istituto Zooprofilattico Sperimentale della Lombardia e dell’Emilia-Romagna “Bruno Ubertini”, 42124 Brescia, Italy; c.lucchetti07@gmail.com (C.L.); mara.scremin@izsler.it (M.S.); mattia.calzolari@izsler.it (M.C.); deborah.torri@izsler.it (D.T.); paolo.bonilauri@izsler.it (P.B.); michele.dottori@izsler.it (M.D.); 2 Dipartimento di Scienze Mediche Veterinarie, Alma Mater Studiorum Università di Bologna, 40064 Ozzano dell’Emilia, Italy; federica.savini3@unibo.it

**Keywords:** *Piophila casei*, Parma ham, growth, ADD, isomegalen-diagram, isomorphen-diagram

## Abstract

The cheese (or ham) skipper, *Piophila casei* (Linnaeus), belongs to the family Piophilidae and is a detritivore species that feeds on decaying organic matter. Its larvae are commonly found on high-protein substrates (including carcasses), with a life cycle that is strongly influenced by environmental temperature and food nature. In this study, we investigated development time and larval length of *P. casei* reared on Parma ham PDO (Protected Designation of Origin) at five constant temperatures (20, 22, 24, 26, 28 °C). We calculated the accumulated degree days (ADD) and constructed isomegalen- and isomorphen-diagrams to estimate the infestation time. The minimum development threshold (t_L_) obtained by extrapolating the development rates measured between 20 °C and 28 °C was 9.91 °C, resulting in ADDs (oviposition–eclosion) of 216.9, 219.9, 220.7, 209.2, and 220.1 at 20, 22, 24, 26, and 28 °C, respectively.

## Simple Summary

Cured meats are prone to infestation by various insects, including the skipper fly *Piophila casei*. This small fly can damage food by tunneling into it and may act as a vector for pathogens, making the infested food unsuitable for consumption. Specifically, in ham, females lay eggs, and the newly emerged larvae feed on the product. The development time of *P. casei* is influenced by several factors, primarily temperature and the nature of the substrate. Determining the onset of an infestation is crucial for its effective management in terms of prevention throughout the shelf life and market life of the product. In our study, we reared *P. casei* on cured ham at five different temperatures and calculated the corresponding development time by means of two analytical approaches, namely accumulated degree days (ADDs) and isomegalen-/isomorphen-diagrams. These methods allow for the estimation of insect age based on accumulated thermal units, body length, or developmental stage.

## 1. Introduction

In Italy, the food industry represents a major economic sector, with a total value of approximately EUR 132 billion, accounting for 8% of the Gross Domestic Product. Within this industry, the ham sector alone is valued at EUR 2.273 billion, underscoring its significant economic importance [1]. Given the high value and cultural relevance of products such as Prosciutto di Parma DOP (PDO, Protected Designation of Origin; hereinafter “Parma ham”), ensuring their safety and quality is essential. *Piophila casei* (Linnaeus) has become a significant concern in ham production, not only due to its destructive feeding habit [2] but also because the accidental ingestion of larvae can lead to enteric myiasis, posing a public health risk [3,4].

*P. casei* is a member of the Piophilidae family, a small group of flies within the Tephritoidea superfamily, which is distributed worldwide [5]. Females can mate 24 h after the emergence, beginning oviposition about 10 h after mating, enabling rapid population growth by laying up to 250 eggs [6]. The eggs hatch quickly, and the larvae feed on the substrate, completing their development in just a few days depending on temperature and food source. Mature larvae exhibit a unique escape behavior (hence the name “skipper”) of propelling themselves away from the substrate by forming a U-shape and releasing, allowing them to spread to adjacent products [7].

In the ham production process, the curing phase of dry cured ham is the most critical phase, since hams are placed in close proximity in large ventilated rooms at a controlled temperature, where the infestation can quickly pass from one ham to another, completing five to six generations per year [8]. Not only is it difficult to distinguish infested hams from others, but the larvae are also very resistant to temperature variations, being capable of surviving within a range from 51 °C to −15 °C. In addition, fully grown larvae can survive for 6 months without any food [9]. However, ham can also become infested in the stages following production, namely during storage before/after sale.

*P. casei* has proven to be a useful tool in the forensic investigation of post-mortem remains; specifically, forensic entomologists have relied on the presence of *P. casei* larvae to assist in the estimation of time of death for human remains, since the larvae are usually found on the human body after active decay, when the corpse begins to dry [10]. Entomologists utilize knowledge of the current instar of the collected larvae, coupled with measurements of weather and temperature conditions, to provide an estimation of the post-mortem interval (PMI). The substrate significantly influences the development rate of insects [11] such as *P. casei* larvae. This information is crucial when estimating the PMI or the date of egg laying, especially in forensic entomology or food safety investigations.

Indeed, the same approach can be applied to date the infestation of a food product, in order to identify its origin. This is essential for implementing effective monitoring and control strategies, as it can help minimize economic losses due to product spoilage and disposal.

This study aims to evaluate the development of *P. casei* on Parma ham at five constant temperatures. The present work focuses on temperatures above 20 °C, which are more representative of post-production storage conditions. This approach allows for the development of a predictive model that can be applied to retail scenarios, where early detection of infestation is particularly challenging. We calculated accumulated degree days (ADDs) and constructed isomegalen- and isomorphen-diagrams to estimate infestation timing. These tools can support both forensic investigations and food safety assessments.

## 2. Materials and Methods

Adults and larvae of *Piophila casei* were derived from infested hams obtained from a facility facing critical infestation issues. Adults were identified using the morphological characteristics described by Rochefort, Iwasa and McAlpine [12,13]. About 200 larvae were transferred into plastic containers (27 cm in length, 17 cm in width, and 15 cm in height) with vermiculite and supplied with about 80 g of artificial diet. The artificial diet was composed of milk powder (80 g), dry yeast (50 g), agar (14 g), ethyl alcohol (10 mL), and water (1000 mL) [14]. The diet was employed exclusively for maintaining the laboratory colony and was replenished daily until the pupal stage was reached. Emerged adults were transferred into a mating cage (30 cm in length, width, and height) containing a cotton pad soaked with a saturated glucose solution. The colony was maintained in a growth chamber at 25 °C with 70% RH and a photoperiod of 12:12 (L:D).

### 2.1. Length Data Analysis

A piece of ham (Prosciutto di Parma DOP, aged for 3 months) weighing about 50 g was placed in the mating cage to induce egg laying. The ham was checked under a stereomicroscope every 2 h and, as soon as about 200 eggs were laid, transferred into a plastic container (27 cm in length, 17 cm in width, 15 cm in height) with vermiculite. The containers were placed in a precision climatic chamber at 20, 22, 24, 26, 28 ± 1 °C, with 70% ± 5 RH and a photoperiod of 12:12 (L:D). A 50 g sample of fresh ham was added every 2 days. Each day at the same time, 10 larvae were placed in hot water (<80 °C) and maintained for 30 s according to best practices in forensic entomology [15]. They were then transferred to a Petri dish, photographed, and measured to construct isomegalen-/isomorphen-diagrams. At the same time, a holder instar was recorded for the minimum development threshold (t_L_) and ADD calculation. Six replicates were made for each tested temperature.

Each larva was photographed under 40× magnification with the software NIS-Elements V.4.0 (Kanagawa, Japan) connected to a digital camera. Larvae were measured individually using the software ImageJ 1.46r (Bethesda, MD, USA); length measurement was performed daily by a single operator to reduce inter-operator variability [16], starting on the day the eggs hatched and stopping when 10% of the sample underwent pupation. Pupation was defined by instar identification.

### 2.2. Lower Developmental Threshold and Accumulated Degree Days

The lower developmental threshold temperature (t_L_) was estimated using linear regression analysis. Developmental rates, calculated as the inverse of developmental time (i.e., y = 1/developmental time), were plotted against constant temperatures (x). A linear regression line was fitted to these data points. From the resulting regression equation, the lower threshold temperature (t_L_) was determined by extrapolating the line to the point where the developmental rate equals zero (i.e., y = 0). This corresponds to the x-intercept of the regression line [17].

The accumulated degree days from egg to pupa and from egg to eclosion were calculated for each replicate (n = 6) and for each constant temperature regimen, according the formula

ADD = ∑d(t − t_L_)

where d (days) is the development time and t (temperature) is the rearing temperature.

### 2.3. Statistical Analysis

Larval length and development time were reported as mean ± standard deviation values. One-way ANOVA revealed statistically significant differences in developmental time across temperatures (*p* < 0.001), confirming that development is temperature-dependent; post hoc HSD tests showed that most pairwise comparisons were significant (*p* < 0.05) except between 26 and 28 °C, which did not differ significantly at any developmental interval. The assumptions of normality and homoscedasticity were assessed using Bartlett’s test for equal variances.

A comparison of growth curves (e.g., testing for differences in shape, slope, or intercept of growth curves) across the five constant temperatures was conducted following the methodology described by Rao [18].

## 3. Results

Development times from oviposition to pupariation and from oviposition to eclosion are presented in Table 1. The duration of each developmental time decreases progressively with increasing temperature. Specifically, the mean time from oviposition to eclosion was 21.5 ± 0.5 days at 20 °C and 12.2 ± 0.4 days at 28 °C. One-way ANOVA revealed statistically significant differences in developmental time across temperatures (*p* < 0.001), confirming that development is temperature-dependent.

Post hoc comparisons using the HSD test indicated that most pairwise differences between temperatures were statistically significant (*p* < 0.05), with the exception of the comparison between 26 and 28 °C, which was not significant at any developmental interval. These results suggest that while development accelerates with increasing temperature, the rate of change diminishes at higher temperatures.

### 3.1. Analysis of Accumulated Degree Days

**Figure 1:**
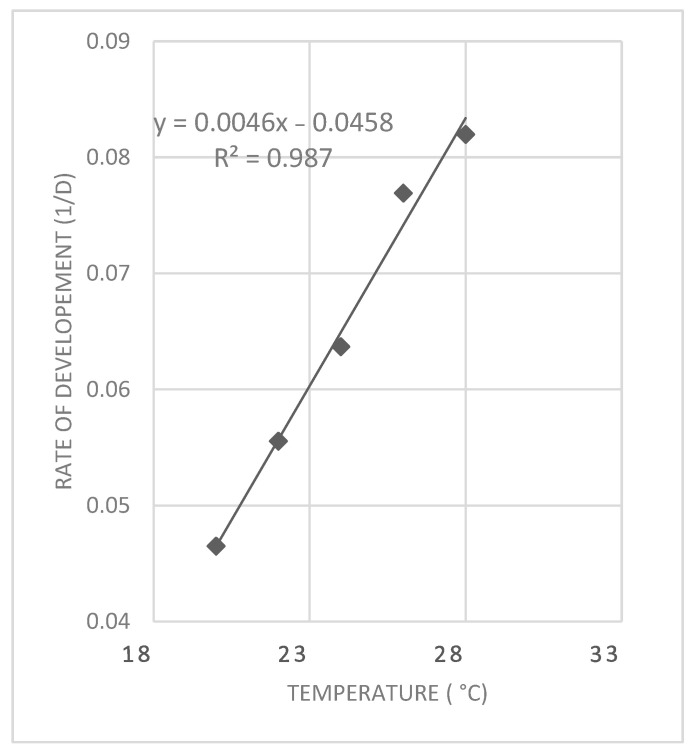
Linear relationship between temperature and development rate (1/days) of *P. casei* from oviposition to adult eclosion. Each point represents the mean development rate at a given constant temperature. The regression analysis yielded a high coefficient of determination (R^2^ = 0.98), indicating a strong linear fit.

Based on the observed duration of development at each temperature, the minimum development threshold (t_L_) was estimated by linear interpolation of development rate (1/days) against temperature (Figure 1). A strong linear relationship was observed between temperature and development rate in the range between 20 °C and 28 °C (R^2^ = 0.98). The estimated lower developmental threshold (t_L_) for *P. casei* was 9.91 °C.

Statistical analysis of ADD from oviposition to pupariation and from oviposition to eclosion revealed no significant differences among the five constant temperatures tested (*p* > 0.05). Post hoc comparisons using Tukey’s HSD test confirmed the absence of statistically significant pairwise differences. These results suggest that ADD values exhibit relative stability across the tested temperature range and conditions, as expected.

Based on the observed developmental durations and the t_L_, the ADDs were calculated for each temperature and replicate (n = 6) both from oviposition to pupariation and from oviposition to adult eclosion. The results are presented in Table 2.

### 3.2. Isomegalen- and Isomorphen-Diagram

The mean larval lengths (±standard deviations) of *P. casei* at five constant temperatures are presented in Table 3. The minimum and maximum mean lengths recorded were 0.84 and 8.53 mm, respectively. As temperature increased, the developmental time decreased. However, a comparison of growth curves across the five temperatures—evaluating differences in shape, slope, and intercept—did not reveal statistically significant differences. Pairwise comparisons of mean larval lengths showed significant differences only between 28 °C and 20 °C or 22 °C, whereas no significant differences were observed between 24 °C and 28 °C or between 20 °C and 26 °C.

**Figure 2:**
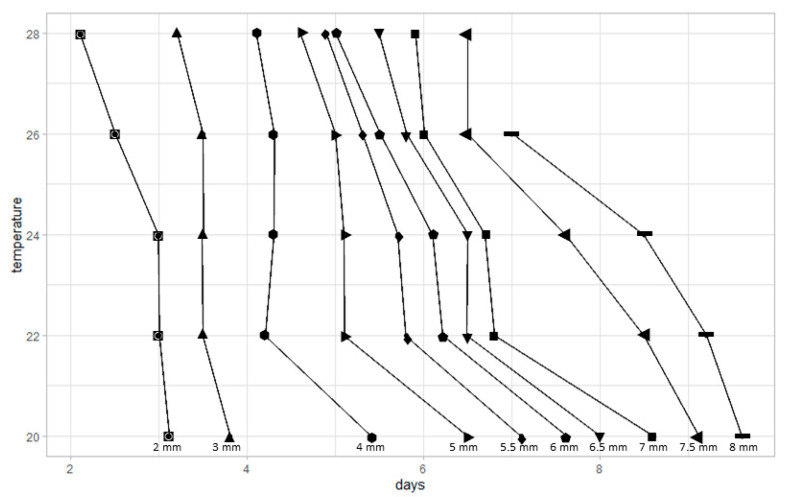
Isomegalen-diagram of *P. casei* at five constant temperatures. Each curve represents the mean larval growth over time. Time points were derived from the mean duration of development from oviposition to pupariation at each temperature. “◙” denotes isomegalen 2 mm, “▲” denotes isomegalen 3 mm, “⬢” denotes isomegalen 4 mm, “►” denotes isomegalen 5 mm, “♦” denotes isomegalen 5.5 mm, “⬟” denotes isomegalen 6 mm, “▼” denotes isomegalen 6.5 mm, “■” denotes isomegalen 7 mm, “◄” denotes isomegalen 7.5 mm, and “▬” denotes isomegalen 8 mm.

Larval growth data were used to construct an isomegalen-diagram for *P. casei* at five constant temperatures (Figure 2). The diagram displays ten isomegalen curves, each representing a constant larval length plotted against time. For each temperature, the time required to reach a specific larval length was estimated based on the average developmental duration from oviposition to pupariation. The resulting curves illustrate the temperature-dependent dynamics of larval growth, with faster development observed at higher temperatures.

**Figure 3:**
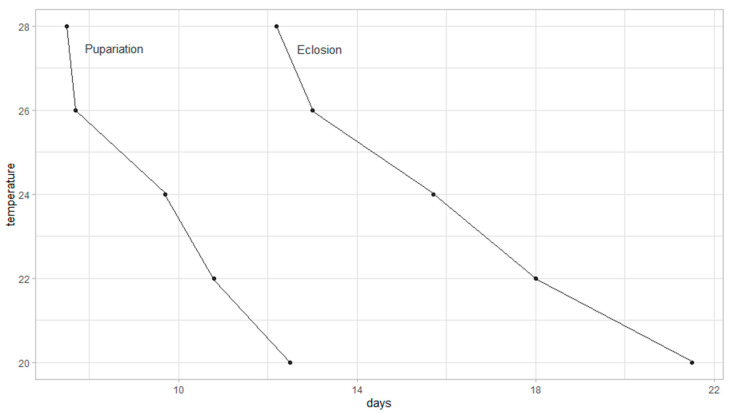
Isomorphen-diagram of *P. casei* at five constant temperatures. Each curve represents the mean time required to reach specific morphological stages (pupariation and eclosion) from oviposition.

Developmental data from oviposition to pupariation and from oviposition to adult eclosion were used to construct an isomorphen-diagram (Figure 3). Each curve represents the timing of identical morphological stages across five constant temperatures. The diagram illustrates how increasing temperature accelerates development, with shorter durations observed at higher temperatures for both pupariation and eclosion.

## 4. Discussion

In food production and storage environments, insect infestation can occur at various stages, potentially compromising product safety and quality. Similar to forensic investigation, entomological evidence can be employed to estimate the timing of infestation [19], thereby identifying the critical point in the production chain at which contamination occurred. The substrate on which the insect larvae feed plays a critical role in determining their developmental rate [20]; for species such as *Piophila casei*, which is commonly found in cured meats like ham, the nutritional and physical properties of the substrate can significantly alter the timing of larval growth stages.

This variability is particularly relevant when estimating the date of egg laying in food contamination assessments. Using developmental data derived from substrates that differ from the actual infestation context can lead to inaccurate conclusions. Therefore, it is essential to use substrate-specific developmental data that closely mimic the real-world conditions under which the infestation occurs. Our study aims to evaluate the development of *P. casei* on Parma ham at five constant temperatures. This study employs a dual approach to estimate the infestation time of ham by *P. casei*, namely using the accumulated degree days (ADD) and isomegalen/isomorphen-diagrams based on the achievement of length/stadium at five constant temperatures. When possible, both methods should be used in combination to improve accuracy. If the larvae are alive, they can be reared until the term of development (adult emergence) at a constant temperature, and the ADDs can be used retrospectively to estimate the oviposition window.

Alternatively, larval length can be measured and compared to isomegalen curves to estimate age. If pupae are found, isomorphen-diagrams are more appropriate.

Our results show that the ADD values remained relatively stable across the tested temperature range, suggesting that this method is robust for estimating infestation timing. However, larval length comparisons and growth curves across temperatures revealed fewer statistical differences, especially at lower temperatures. This indicates that isomegalen-diagrams should be interpreted with caution under such conditions. Moreover, the absence of significant differences between 26 °C and 28 °C in developmental time (both intervals) may reflect a plateau in the development rate. To confirm this hypothesis, however, additional experiments at temperatures above 28 °C would be necessary.

Our findings further emphasize the critical role of the rearing substrate in insect development. Compared to previous studies that used artificial diets, the larvae reared on Parma ham developed significantly faster. For instance, Russo [21] reported a development time of 19.7 days from oviposition to pupariation at 28 °C, whereas our study observed a markedly shorter duration of 7.5 days under the same temperature conditions. This discrepancy may be attributed to both nutritional factors (i.e., substrate composition) and chemical–physical properties (such as texture and humidity) [22,23], highlighting the importance of using realistic substrates when modeling insect development for applied purposes.

Another study reported the development times of *P. casei* reared on pork, a substrate closely resembling the one used in our experiment [24]. A comparison between the datasets shows strong agreement, reinforcing the reliability of our observations. At 20 °C, our study recorded a development time of 21.5 days, whereas the comparative study reported 23.7 days. At higher temperatures, the development times were even closer: 12.2 days in our case versus 12.7 days in the reference study.

These results confirm the importance of the rearing substrate in the application of accumulated degree days (ADDs) and isomegalen/isomorphen-diagrams for accurately dating infestations.

The preliminary results of this study were presented at the SIDILV Conference 2023, Brescia, Italy, 11–13 October 2023 [25].

## 5. Conclusions

If food is found to be infested by *Piophila casei*, the infestation time can be estimated by knowing the storage temperature and food origin. Determining the onset of the infestation is crucial, as it enables more effective monitoring and control strategies and helps reduce economic losses associated with product spoilage and disposal.

This study provides new insights into the developmental biology of *P. casei*, with a specific focus on its growth on cured ham—a substrate not previously considered in the literature. In particular, the data can be used to estimate the onset of food infestation, thereby supporting timely and targeted intervention strategies in food safety and pest management contexts.

Temperature is also a critical factor: future studies should aim to include lower temperature ranges to better represent the full spectrum of conditions encountered during the maturation and storage phases of cured meats.

## Figures and Tables

**Table 1:** Mean ± standard deviation of developmental time (in days) of *Piophila casei* from oviposition to pupariation and from oviposition to adult eclosion at five constant temperatures. Different superscript letters indicate statistically significant differences (*p* < 0.05).

Stage	20 °C	22 °C	24 °C	26 °C	28 °C
Oviposition–pupariation	12.5 ± 0.5 ^a^	10.8 ± 1 ^b^	9.7 ± 0.8 ^c^	7.7 ± 0.8 ^d^	7.5 ± 0.5 ^d^
Oviposition–eclosion	21.5 ± 0.5 ^a^	18 ± 0.9 ^b^	15.7 ± 0.8 ^c^	13 ± 0.6 ^d^	12.2 ± 0.4 ^d^

**Table 2:** Accumulated degree days (ADDs) of *P. casei* at five constant temperatures from oviposition to pupariation and from oviposition to adult eclosion of six replicates. Values are expressed as ADD mean ± standard deviation.

Stage	20 °C	22 °C	24 °C	26 °C	28 °C
Oviposition–pupariation	126.1 ± 5.5	131.0 ± 11.9	136.2 ± 11.5	123.4 ± 13.1	135.7 ± 9.9
Oviposition–eclosion	216.9 ± 5.5	219.9 ± 6.9	220.7 ± 11.5	209.2 ± 10.2	220.1 ± 7.4

**Table 3:** Means of larval length (mm (±standard deviations)) of *P. casei* at five temperatures. “*”denotes a missing value because the insect had already changed morphological stage.

Days	20 °C	22 °C	24 °C	26 °C	28 °C
1	1.17 ± 0.02	0.84 ± 0.02	1.07 ± 0.15	1.14 ± 0.14	1.144 ± 0.20
2	1.28 ± 0.15	1.44 ± 0.11	1.54 ± 1.53	1.71 ± 0.25	1.92 ± 0.64
3	1.77 ± 0.27	2.09 ± 0.33	2.1 ± 0.00	2.22 ± 0.14	2.91 ± 1.38
4	3.42 ± 0.88	3.86 ± 0.08	3.47 ± 0.00	3.45 ± 0.20	3.77 ± 1.04
5	3.50 ± 0.88	5.06 ± 0.77	4.90 ± 0.38	4.98 ± 1.03	6.03 ± 1.31
6	4.55 ± 0.87	5.63 ± 1.68	5.93 ± 1.09	7.06 ± 1.10	7.14 ± 1.56
7	5.40 ± 0.79	7.15 ± 1.25	7.49 ± 0.90	8.06 ± 0.77	7.61 ± 0.50
8	6.42 ± 0.82	7.22 ± 0.70	7.87 ± 0.66	8.22 ± 0.66	*
9	7.38 ± 0.48	7.68 ± 0.56	8.07 ± 0.08	*	*
10	8.27 ± 0.10	8.53 ± 0.32	*	*	*
11	8.17 ± 0.00	*	*	*	*

## Data Availability

The original contributions presented in this study are included in the article. Further inquiries can be directed to the corresponding author.
